# Sibutramine Effects on Central Mechanisms Regulating Energy Homeostasis

**DOI:** 10.2174/157015912799362788

**Published:** 2012-03

**Authors:** João R Araújo, Fátima Martel

**Affiliations:** Department of Biochemistry (U38-FCT), Faculty of Medicine of Porto, University of Porto, Porto, Portugal

**Keywords:** Appetite, obesity, sibutramine, hypothalamus, weight loss.

## Abstract

During the last 50 years the global pandemic of obesity and associated life-threatening co-morbidities strongly promoted the development of anti-obesity pharmacotherapy. Sibutramine is an anti-obesity drug that in conjunction with lifestyle modifications reduces food intake and body weight. This may result from several effects: inhibition of presynaptic reuptake of monoaminergic neurotransmitters in the central nervous system, thereby suppressing appetite, induction of an increase in anorexigenic and a decrease in orexigenic neuropeptide secretion, induction of an increase in energy expenditure, and induction of peripheral sympathomimetic effects. The effects of sibutramine on anabolic and catabolic signals that regulate energy homeostasis in the hypothalamus are not completely understood. So, the aim of this review is to summarize the central mechanisms of action of sibutramine, responsible for its weight and food intake reducing potential. Despite being a useful drug in obesity treatment, awareness about the loss of long-term effectiveness and detrimental side effects of sibutramine has recently emerged. As a consequence, new drugs that produce safer and more persistent weight loss are currently undergoing clinical trials.

## INTRODUCTION

Obesity is a chronic disease that can be defined as an excessive accumulation of body fat [1]. Its prevalence is increasing worldwide and nowadays more than 300 million individuals are obese and an additional 800 million are pre-obese or overweight. Obesity, particularly central obesity, increases the risk of cardiovascular (CDV) and cancer mortality, thereby reducing life expectancy [2]. Lack of effective weight-loss treatments based solely on lifestyle modifications (diet and physical exercise), promoted the development of anti-obesity drugs as an adjunctive treatment. Patients with a body mass index (BMI) persistently exceeding 30 kg/m^2^ or with a BMI ≥ 27kg/m^2^ with concomitant obesity-related co-morbidities (e.g. coronary heart disease, hypertension, type 2 diabetes, dyslipidemia or obstructive sleep apnea), are eligible for anti-obesity drug treatment [3, 4]. The mechanisms of action of anti-obesity drugs that lead to weight loss include: (a) a reduction of food intake and appetite by amplifying anorexigenic (i.e. suppressors of food intake) or blocking orexigenic (i.e. stimulators of food intake) afferent and efferent central nervous system (CNS) signals, (b) a decrement of body weight reference value (body weight set point) determined by the hypothalamic areas that control energy homeostasis, (c) an increase in energy expenditure, (d) a decrease in nutrient (especially fat and carbohydrates) digestion and intestinal absorption, and (e) a reduction of fat storage and/or increase of hydrolysis of triglycerides in adipose tissue [1, 4, 5]. During the last 50 years, CNS energy regulatory mechanisms that control appetite, food intake and energy expenditure have been the target of most of the developed anti-obesity drugs [6]. Sibutramine, is one of these drugs. So, the aim of this review is to summarize the effects of sibutramine upon the mechanisms controlling energy homeostasis, which are responsible for its weight reduction potential. Additionally, sibutramine long-term effectiveness and side effects will be briefly described. 

## NEUROENDOCRINE MODULATION OF APPETITE 

**Fig. (1):**
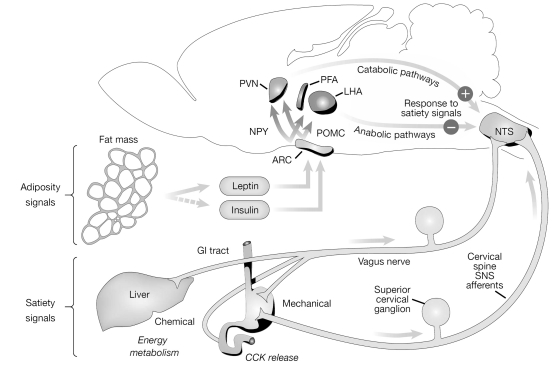
Neuroanatomical model summarizing the different pathways that control energy homeostasis. During meals, episodic and tonic signals, such as the satiety signals cholecystokinin (CCK) and the adiposity signals leptin and insulin, are conveyed through the vagus nerve to the arcuate nucleus (ARC) and nucleus of the solitary tract (NTS). These signals interact with neurons that synthesize anorexigenic and orexigenic neuropeptides, like proopiomelanocorticotropin (POMC)/cocaine amphetamine regulating transcript (CART) or neuropeptide Y (NPY)/agouti related peptide (AgRP), respectively, which in turn project to other hypothalamic areas including the paraventricular nuclei (PVN) and the lateral hypothalamic area (LHA). Figure reproduced, with permission, from Reference [5]. Abbreviations: GI, gastrointestinal; PFA, perifornical area; SNS, sympathetic nervous system.

Appetite is the desire for food intake. Its regulation relies on the integration and transduction of episodic and tonic signals in the CNS energy regulatory centers located in the hypothalamus, particularly in the arcuate nucleus (ARC) [6, 7]. Episodic signals are short-term inputs generated before meal intake, while tonic signals are short- or long-term inputs generated by food consumption, absorption and metabolism and body energy stores. Both can be anabolic (stimulating energy intake and suppressing energy expenditure) or catabolic (inhibiting energy intake and stimulating energy expenditure). Tonic and episodic signals can be divided into three categories: 1) signals originating from stimulation of receptors in the gut and from metabolic changes in the liver and adipose tissue, which are conveyed *via *vagal afferent signals or hormones to receptors located in the ARC and in the nucleus of the solitary tract/area postrema complex in the brain stem. These can be satiety signals (e.g. anabolic and catabolic gastrointestinal peptides like ghrelin and cholecystokinin, respectively) and adiposity signals (e.g. leptin and insulin); 2) nutrients and neurotransmitter precursors that cross the blood brain barrier altering CNS neurochemical activity, and 3) in response to these two inputs, efferent signals that arise from receptors located in the CNS detecting circulating levels of nutrients, hormones and other factors of the periphery [6, 8, 9]. These efferent signals include numerous orexigenic and anorexigenic neuropeptides and monoaminergic neurotransmitters, that are contained in neurons which project from the ARC to downstream areas involved in the perception of satiety (ventromedial nuclei and paraventricular nuclei (PVN)) and hunger (lateral hypothalamic area (LHA)) [10]. The net output of the PVN is catabolic, enhancing the potency of satiety signals, whereas the net output of the LHA is by contrast anabolic, suppressing the activity of the satiety signals (see Fig. **1**). Taken together, fluctuations in episodic and tonic signals strongly determine appetite, hunger and satiety being thus critical for body weight regulation [6].

## PHARMACOLOGICAL TARGETS OF SIBUTRAMINE 

Sibutramine, with the trade names Meridia^®^ and Reductil^®^, is an orally administered drug currently approved in the United States of America, and until recently also approved in Europe, for the long-term treatment of obesity [11]. It is a *β*-phenethylamine [4] that, when administered in doses of 5-15 mg/day and in conjunction with lifestyle therapy (reduced caloric diet, physical exercise and eating behavior therapy), can induce a modest but significant 5–10% body weight loss (4-8 kg) in most obese and overweight patients [2, 4, 5, 12]. The ability of sibutramine to reduce body weight can be explained by several reasons. First, its most well described effect is a selective inhibition of presynaptic reuptake of the monoaminergic neurotransmitters serotonin (5-hydroxytryptamine; 5-HT), noradrenaline (NA) and, to a lesser extent, dopamine, at the CNS level, enhancing the appetite suppressing actions of these neurotransmitters [13]. Reuptake of these neurotransmitters from the synapse *via *high affinity and specific transporters present in the presynaptic membrane (which are inhibited by sibutramine), terminates its signaling [14]. Unlike the early amphetamine-like drugs such as dexamphetamine, phentermine and fenfluramines, sibutramine does not directly stimulate the release of 5-HT, NA nor dopamine, and so does not causes presynaptic neurotransmitters depletion nor the consequent neurotoxicity [15]. Moreover, there is evidence that sibutramine does not bind to any of the serotoninergic, adrenergic or dopaminergic postsynaptic receptors [16]. Second, sibutramine increases anorexigenic and reduces orexigenic neuropeptides release in the ARC. Studies in animal models have contributed to better elucidate this effect. Indeed, in energy-restricted rats, sibutramine treatment increased the transport of the anorexigenic hormone leptin into the ARC, which in turn activated proopiomelanocorticotropin (POMC)/cocaine amphetamine regulating transcript (CART) neurons and inhibited neuropeptide Y (NPY)/agouti related peptide (AgRP) neurons [8, 16-18]. Consequently, this stimulated the secretion of the anorexigenic neuropeptides POMC and its derived melanocortin α-melanocyte-stimulating hormone (*α*-MSH), and of corticotrophin-releasing hormone (CRH) and CART [8, 16, 18]. Released* α*-MSH may bind to and activate melanocortin receptors (MCR), particularly MCR-4, in the PVN [7]. On the contrary, the secretion of the orexigenic neuropeptides NPY, AgRP, orexin A and B and melanocortin-concentrating hormone (MCH) is inhibited [8, 18]. 

The ability of sibutramine to decrease body weight can also be attributed to the fact that it increases energy expenditure. This can be achieved by two distinct effects. Firstly, sibutramine prevents the decrease of basal energy expenditure that follows weight loss [2], an effect than can be explained by activation of MCR-4 [7]. Secondly, sibutramine may increase thermogenesis [5] trough *β*_3_-adrenergic receptor activation, an effect mediated by NA, in peripheral white adipose tissue [16]. Additionally, in rats but not in humans, sibutramine seems to induce hypophagia through sympathomimetic effects, involving down-regulation of presynaptic and postsynaptic *β*_1_- and *α*_2_-adrenergic and 5-HT_1A_ receptors located in the PVN. These effects are known to be associated with an antidepressant action [16, 18], which explains why sibutramine was originally developed as an antidepressant drug [13]. However, sibutramine is nowadays used as an appetite suppressant agent, being until recently the most widely prescribed one [18]. Weight-reducing effects of sibutramine are largely attributed to its active primary (N-desmethylsibutramine; BTS 54505) and secondary (N-didesmethylsibutramine; BTS 54 354) amine metabolites rather than to the parent compound [1]. 

## SIBUTRAMINE LONG-TERM EFFECTIVENESS

Normally, 6 months after the initiation of sibutramine treatment food intake recovers and body weight plateaus until sibutramine is discontinued [1]. This phenomenon is called sibutramine tachyphylaxis. Upon discontinuation, regain of the lost weight is frequently observed. This highlights that sibutramine is effective at reducing body weight and food intake when given for short periods of time (6-12 months), but its effectiveness diminishes when administered chronically (> 1 year) [18]. Indeed, in humans there are no studies longer than 2 years evaluating the weight reducing effects of sibutramine [2]. Sibutramine tachyphylaxis may be explained by homeostatic counterregulatory mechanisms that promote resistance to further weight reduction actions of this drug [4]. These counterregulatory mechanisms can be explained as follow. Short-term administration of sibutramine lowers the body weight set point. This body weight set point is not permanently reduced by long-term administration of sibutramine, since the brain will oppose this change by stimulating appetite [8], food reward (pleasure associated with consumption of a palatable food) [10, 18] and food intake, and also by decreasing basal energy expenditure and dampening reductions in body fat stores. Boozer *et al.* [19] points out that the decreased circulating levels of leptin in blood accompanying body fat reduction may trigger counter regulatory mechanisms that block further weight loss effects of sibutramine, as they found out that restoration of circulating levels of leptin during long-term (8-weeks) sibutramine therapy decreased body weight and food intake and increased fat oxidation in obese rats [8]. 

## SIBUTRAMINE SIDE EFFECTS 

Despite the short-term efficacy in reducing body weight and food intake, sibutramine has some undesirable side-effects which may or may not be directly related with its anti-obesity effect. The main side effects of sibutramine are associated with its noradrenergic stimulation of hypothalamic appetite circuits and with its inherent sympathomimetic properties. Dry mouth, headache, insomnia, asthenia, obstipation [5] and in some cases amnesia [20] are some of the side effects. Sibutramine can also induce mood changes because control of energy homeostasis and of mood often use overlapping brain circuits (e.g. the serotoninergic system) (11). Contrary to other appetite-suppressing drugs (e.g. phentermine and dexamphetamine) and despite being structurally similar to amphetamine, sibutramine has no abuse potential since it does not enhance dopamine release at the synapse [16]. Due to an increased sympathetic drive to the CDV system, sibutramine can rise systolic and/or diastolic blood pressure (2–20 mm Hg) and heart rate (3–20 beats/min) [13]. In agreement with these effects, the Sibutramine in Cardiovascular Outcomes Trial (SCOUT) found that in obese and overweight subjects, sibutramine increased morbidity from CDV disease (myocardial infarction or stroke) [18], leading the European Medicines Agency to withdrawal this drug from the market in 2010 [13]. So, because of its inherent cardiovascular side effects, sibutramine cannot be used to test the hypothesis that weight loss can decrease cardiovascular risk. In Europe, this leaves just one approved drug – Orlistat^®^ - for the long-term treatment of obesity [13].

## CONCLUSIONS

Sibutramine is an anti-obesity drug that produces short-term and modest body weight reductions mainly due to its appetite-suppressing effect. Despite being a useful drug in obesity treatment, awareness about the loss of effectiveness and detrimental side effects of sibutramine has recently emerged. As a consequence, the identification of new drugs that safely and persistently induce weight loss is currently an intense field of research [4, 5]. Drugs that target orexigenic and anorexigenic neuropeptides release such as MCR-4 agonists, MCH receptor antagonists and NPY antagonists, and others that target monoaminergic neurotransmitters receptors such as 5-HT_2C_ serotonin receptor agonists are, among others, currently undergoing clinical trials [4].
